# Incidence of SARS-CoV-2 Infection, Emergency Department Visits, and Hospitalizations Because of COVID-19 Among Persons Aged ≥12 Years, by COVID-19 Vaccination Status — Oregon and Washington, July 4–September 25, 2021

**DOI:** 10.15585/mmwr.mm7046a4

**Published:** 2021-11-19

**Authors:** Allison L. Naleway, Holly C. Groom, Phil M. Crawford, S. Bianca Salas, Michelle L. Henninger, Judy L. Donald, Ning Smith, Mark G. Thompson, Lenee H. Blanton, Catherine H. Bozio, Eduardo Azziz-Baumgartner

**Affiliations:** ^1^Center for Health Research, Kaiser Permanente Northwest, Portland, Oregon; ^2^CDC COVID-19 Response Epidemiology and Surveillance Task Force; ^3^Influenza Division, National Center for Immunization and Respiratory Diseases, CDC.

Population-based rates of infection with SARS-CoV-2 (the virus that causes COVID-19) and related health care utilization help determine estimates of COVID-19 vaccine effectiveness and averted illnesses, especially since the SARS-CoV-2 B.1.617.2 (Delta) variant began circulating in June 2021. Among members aged ≥12 years of a large integrated health care delivery system in Oregon and Washington, incidence of laboratory-confirmed SARS-CoV-2 infection, emergency department (ED) visits, and hospitalizations were calculated by COVID-19 vaccination status, vaccine product, age, race, and ethnicity. Infection after full vaccination was defined as a positive SARS-CoV-2 molecular test result ≥14 days after completion of an authorized COVID-19 vaccination series.* During the July–September 2021 surveillance period, SARS-CoV-2 infection occurred among 4,146 of 137,616 unvaccinated persons (30.1 per 1,000 persons) and 3,009 of 344,848 fully vaccinated persons (8.7 per 1,000). Incidence was higher among unvaccinated persons than among vaccinated persons across all demographic strata. Unvaccinated persons with SARS-CoV-2 infection were more than twice as likely to receive ED care (18.5%) or to be hospitalized (9.0%) than were vaccinated persons with COVID-19 (8.1% and 3.9%, respectively). The crude mortality rate was also higher among unvaccinated patients (0.43 per 1,000) than in fully vaccinated patients (0.06 per 1,000). These data support CDC recommendations for COVID-19 vaccination, including additional and booster doses, to protect individual persons and communities against COVID-19, including illness and hospitalization caused by the Delta variant (*1*).

As of November 15, 2021, SARS-CoV-2 had infected approximately 46 million persons in the United States and caused approximately 759,000 deaths (*2*). A surge in cases, hospitalizations, and deaths began in June 2021 with the emergence of the Delta variant; after July 4, Delta became the predominant lineage in the U.S. Pacific Northwest (*3*). As of November 15, approximately 68% of the U.S. population had received ≥1 dose of an authorized COVID-19 vaccine, and approximately 59% of the population was fully vaccinated (*4*). To understand what percentage of authorized COVID-19 vaccine recipients developed infection resulting in ED visits or hospitalizations compared with unvaccinated persons, the incidence and characteristics of illness in vaccinated and unvaccinated persons with SARS-CoV-2 infection were evaluated.

Surveillance for SARS-CoV-2 infection was conducted within Kaiser Permanente Northwest (KPNW), an integrated health care system in Oregon and Washington. Persons aged ≥12 years with continuous health plan enrollment during the July 4–September 25, 2021 surveillance period were included. SARS-CoV-2 infections were identified from nucleic acid amplification test (NAAT) results among symptomatic or asymptomatic persons performed by a KPNW or an affiliated laboratory; rapid antigen tests were not available from KPNW and test results from other settings (e.g., home and school) were not included. Cases were identified through September 11 to permit 2 weeks of follow-up after testing to identify health care utilization.

Vaccination data were obtained from the KPNW electronic medical record, health insurance claims, and the Oregon state immunization information system. Fully vaccinated persons were defined as those with ≥2 doses of an mRNA vaccine product (Pfizer-BioNTech or Moderna) or 1 dose of the Janssen (Johnson & Johnson) vaccine completed ≥14 days before the NAAT. Persons with partial vaccination, defined as receipt of only 1 dose or <14 days since receipt of the second dose of Pfizer-BioNTech or Moderna vaccine, or <14 days since receipt of Janssen vaccine, were excluded (*5*). Unvaccinated persons were those who had no record of COVID-19 vaccination by September 25, 2021.

Age, sex, self-reported race and ethnicity, health care utilization, and underlying medical conditions were obtained from the KPNW electronic medical record. Health care utilization included virtual telephone and video visits, outpatient clinic visits, ED visits, and hospitalizations during the period 3 days before through 14 days after a positive SARS-CoV-2 NAAT test result. Among persons who were hospitalized during the surveillance period, medical records were manually reviewed to ascertain whether the hospitalization was associated with COVID-19, determined by provider notes documenting diagnosis, symptoms, or treatment consistent with COVID-19 (*5*). Information about length of stay, intensive care unit (ICU) admission, and intubation and mechanical ventilation was also abstracted. All records of deaths were also manually reviewed.

Incidence of SARS-CoV-2 infection was calculated by dividing the number of persons with a positive test result by the number of fully vaccinated and unvaccinated persons. Rates were stratified by COVID-19 vaccination status, vaccine product, age, sex, race, and ethnicity, and 95% CIs were calculated assuming the Poisson distribution. Because race and ethnicity were unknown in >10% of the study population, multiple racial groups were combined into a non-White, non-Hispanic category for some analyses. Crude mortality rates were calculated by dividing the number of deaths among persons with a SARS-CoV-2 infection by the number of fully vaccinated and unvaccinated persons. To compare the risk for infection between vaccinated and unvaccinated persons, incidence rate ratios (IRRs) were estimated along with 95% CIs using Poisson regression models with log link function, overall and within demographic subgroups. All analyses were conducted using SAS (version 9.4; SAS Institute). This activity was reviewed by CDC and was conducted consistent with applicable federal law and CDC policy.^†^

Among 482,464 eligible persons identified during the surveillance period, 137,616 (28.5%) were unvaccinated and 344,848 (71.5%) were fully vaccinated. Most (66.5%) vaccinated persons received Pfizer-BioNTech, 27.8% received Moderna, and 5.8% received Janssen.

A total of 7,155 laboratory-confirmed SARS-CoV-2 infections were identified, including 4,146 (57.9%) among unvaccinated and 3,009 (42.1%) among vaccinated persons (Table 1). Overall incidence was 30.1 per 1,000 unvaccinated persons and 8.7 per 1,000 vaccinated persons (IRR = 3.5). IRRs across most strata indicated that incidence was at least three times higher among unvaccinated than among vaccinated persons; IRRs were highest among unvaccinated multiple race persons (4.3), Black persons (4.2), Asian persons (4.1), and adolescents aged 12–17 years (8.9).

**TABLE 1:** Cases of SARS-CoV-2 infections per 1,000 vaccinated and unvaccinated persons — Oregon and Washington, July 4–September 11, 2021

Characteristic	Vaccinated persons*			Unvaccinated persons			IRR (95% CI)**
	Total	No. of cases^†^	Incidence^§^ (95% CI)	Total	No. of cases^†^	Incidence^¶^ (95% CI)	
**Overall**	344,848	3,009	8.7 (8.4–9.0)	137,616	4,146	30.1 (29.2–31.1)	3.45 (3.30–3.62)
**COVID-19 vaccine manufacturer**							
Janssen (Johnson & Johnson)	19,850	303	15.3 (13.6–17.1)	NA	NA	NA	NA
Pfizer-BioNTech	229,216	2,083	9.1 (8.7–9.5)	NA	NA	NA	NA
Moderna	95,782	623	6.5 (6.0–7.0)	NA	NA	NA	NA
**Race/Ethnicity^††^**							
White, NH	238,489	2,155	9.0 (8.7–9.4)	82,308	2,824	34.3 (33.1–35.6)	3.80 (3.59–4.01)
Hispanic	25,993	349	13.4 (12.1–14.9)	10,856	437	40.3 (36.7–44.2)	3.00 (2.61–3.44)
Non-White, NH	41,404	293	7.1 (6.3–7.9)	12,636	435	34.4 (31.3–37.8)	4.86 (4.20–5.63)
Not specified	38,962	212	5.4 (4.8–6.2)	31,816	450	14.1 (12.9–15.5)	2.60 (2.21–3.06)
**Race**							
AI/AN	1,280	18	14.1 (8.9–22.3)	588	23	39.1 (26.0–58.9)	2.78 (1.51–5.11)
Asian	22,828	111	4.9 (4.0–5.9)	3,930	78	19.8 (15.9–24.8)	4.08 (3.06–5.44)
Black/AA	8,224	80	9.7 (7.8–12.1)	4,851	197	40.6 (35.3–46.7)	4.17 (3.23–5.40)
NHPI	1,931	30	15.5 (10.9–22.2)	1,021	63	61.7 (48.2–79.0)	3.97 (2.59–6.09)
White	242,110	2,193	9.1 (8.7–9.4)	83,474	2,862	34.3 (33.1–35.6)	3.79 (3.58–4.00)
All other races	2,142	23	10.7 (7.1–16.2)	848	26	30.7 (20.9–45.0)	2.86 (1.64–4.98)
Multiple races	7,368	59	8.0 (6.2–10.3)	2,054	71	34.6 (27.4–43.6)	4.32 (3.07–6.08)
Not specified	58,965	495	8.4 (7.7–9.2)	40,850	826	20.2 (18.9–21.6)	2.41 (2.16–2.69)
**Ethnicity**							
Hispanic/Latino	25,993	349	13.4 (12.1–14.9)	10,856	437	40.3 (36.7–44.2)	3.00 (2.61–3.44)
Not Hispanic/Latino	278,750	2,439	8.7 (8.4–9.1)	93,994	3,239	34.5 (33.3–35.7)	3.94 (3.74–4.15)
Not specified	40,105	221	5.5 (4.8–6.3)	32,766	470	14.3 (13.1–15.7)	2.60 (2.22–3.05)
**Sex^§§^**							
Female	187,711	1,710	9.1 (8.7–9.6)	63,841	2,074	32.5 (31.1–33.9)	3.57 (3.35–3.80)
Male	156,960	1,299	8.3 (7.8–8.7)	73,592	2,067	28.1 (26.9–29.3)	3.39 (3.17–3.64)
**Age group, yrs**							
12–17	15,234	48	3.2 (2.4–4.2)	15,179	424	27.9 (25.4–30.7)	8.87 (6.58–11.94)
18–24	23,576	228	9.7 (8.5–11.0)	20,817	623	29.9 (27.7–32.4)	3.09 (2.66–3.60)
25–34	46,622	478	10.3 (9.4–11.2)	27,375	903	33.0 (30.9–35.2)	3.22 (2.88–3.59)
35–44	56,291	540	9.6 (8.8–10.4)	23,341	754	32.3 (30.1–34.7)	3.37 (3.02–3.76)
45–54	54,978	561	10.2 (9.4–11.1)	19,885	647	32.5 (30.1–35.1)	3.19 (2.85–3.57)
55–64	57,176	475	8.3 (7.6–9.1)	17,313	481	27.8 (25.4–30.4)	3.34 (2.95–3.79)
65–74	56,607	420	7.4 (6.7–8.2)	9,148	208	22.7 (19.8–26.0)	3.06 (2.60–3.61)
≥75	34,364	259	7.5 (6.7–8.5)	4,558	106	23.3 (19.2–28.1)	3.09 (2.47–3.86)
**Median age, yrs (range)**	**50 (12–104)**	**48 (12–101)**	**NA**	**37 (12–104)**	**36 (12–100)**	**NA**	**NA**

**Abbreviations:** AA = African American; AI/AN = American Indian or Alaska Native; IRR = incidence rate ratio; NA = not applicable; NH = non-Hispanic; NHPI = Native Hawaiian or Other Pacific Islander.

* Received ≥2 doses of Pfizer-BioNTech or Moderna vaccine or 1 dose of Janssen (Johnson & Johnson) COVID-19 vaccine.

^†^ Positive SARS-CoV-2 molecular test result >14 days after second dose of Pfizer-BioNTech or Moderna vaccine dose or first dose of Janssen COVID-19 vaccine.

^§^ Cases per 1,000 vaccinated persons.

^¶^ Cases per 1,000 unvaccinated persons.

** To be more conservative given the large sample size, sensitivity analyses with α levels of 0.01 and 0.005 were conducted, and study findings and conclusions remain unchanged.

**^††^** Persons who self-identified as Hispanic or Latino ethnicity were categorized as Hispanic. Persons who identified as AI/AN, Black, AA, NHPI, multiracial, or any other race were categorized as non-White, NH. The non-White, NH category most commonly included persons who identified as Asian (26,758) or Black (13,075).

^§§^ Persons of unknown sex (177 vaccinated and 183 unvaccinated) are not represented.

Within the vaccinated group, incidence varied by COVID-19 vaccine product received. The highest incidence occurred among Janssen vaccine recipients (15.3 per 1,000), followed by Pfizer-BioNTech (9.1); the lowest incidence was among Moderna recipients (6.5). Vaccinated Hispanic or Latino persons had a higher incidence of SARS-CoV-2 infection (13.4 per 1,000) than did non-Hispanic persons (8.7).

Among unvaccinated persons with SARS-CoV-2 infections, 18.5% had an ED encounter, and 9.0% were hospitalized, compared with 8.1% and 3.9%, respectively, of vaccinated patients (Table 2). Fifty-nine deaths occurred in unvaccinated patients, including 58 who were hospitalized; 22 deaths occurred among fully vaccinated patients, including 21 who were hospitalized. The crude mortality rate among unvaccinated persons (0.43 per 1,000) was sevenfold higher than that among fully vaccinated persons (0.06).

**TABLE 2:** Health care encounters associated with SARS-CoV-2 infections in vaccinated and unvaccinated persons — Oregon and Washington, July 4–September 25, 2021*

Characteristic	Health care encounters, no. (%)							
	Among vaccinated patients (n = 3,009)				Among unvaccinated patients (n = 4,146)			
	Hospitalization	ED visit	Outpatient visit^†^	Virtual visit^§^	Hospitalization	ED visit	Outpatient visit^†^	Virtual visit^§^
**Overall**	**117 (3.9)**	**244 (8.1)**	**862 (28.7)**	**2,696 (89.6)**	**375 (9.0)**	**767 (18.5)**	**1,246 (30.1)**	**3,695 (89.1)**
**COVID-19 vaccine manufacturer**								
Janssen (Johnson & Johnson)	21 (6.9)	33 (10.9)	88 (29.0)	273 (90.1)	NA	NA	NA	NA
Pfizer-BioNTech	81 (3.9)	168 (8.1)	570 (27.4)	1,868 (89.7)	NA	NA	NA	NA
Moderna	15 (2.4)	43 (6.9)	204 (32.7)	555 (89.1)	NA	NA	NA	NA
**Race/Ethnicity^¶^**								
White, NH	94 (4.4)	180 (8.4)	616 (28.6)	1947 (90.3)	269 (9.5)	534 (18.9)	851 (30.1)	2,530 (89.6)
Hispanic	10 (2.9)	27 (7.7)	110 (31.5)	305 (87.4)	30 (6.9)	74 (16.9)	142 (32.5)	396 (90.6)
Non-White, NH	11 (3.8)	28 (9.6)	85 (29.0)	270 (92.2)	54 (12.4)	105 (24.1)	164 (37.7)	394 (90.6)
Unknown	≤5 (—)	9 (4.2)	51 (24.1)	174 (82.1)	22 (4.9)	54 (12.0)	89 (19.8)	375 (83.3)
**Sex**								
Female	63 (3.7)	138 (8.1)	493 (28.8)	1,553 (90.8)	163 (7.9)	383 (18.5)	661 (31.9)	1,884 (90.8)
Male	54 (4.2)	106 (8.2)	369 (28.4)	1,143 (88.0)	212 (10.3)	384 (18.6)	585 (28.3)	1,807 (87.4)
**Age group, yrs**								
12–17	0 (—)	0 (—)	6 (12.5)	41 (85.4)	≤5 (—)	15 (3.5)	84 (19.8)	365 (86.1)
18–24	0 (—)	8 (3.5)	54 (23.7)	195 (85.5)	10 (1.6)	46 (7.4)	150 (24.1)	532 (85.4)
25–34	≤5 (—)	10 (2.1)	131 (27.4)	413 (86.4)	35 (3.9)	104 (11.5)	238 (26.4)	802 (88.8)
35–44	≤5 (—)	24 (4.4)	135 (25.0)	481 (89.1)	39 (5.2)	124 (16.5)	211 (28.0)	677 (89.8)
45–54	12 (2.1)	33 (5.9)	167 (29.8)	495 (88.2)	92 (14.2)	169 (26.1)	234 (36.2)	578 (89.3)
55–64	21 (4.4)	40 (8.4)	131 (27.6)	433 (91.2)	77 (16.0)	144 (29.9)	179 (37.2)	444 (92.3)
65–74	36 (8.6)	67 (16.0)	138 (32.9)	390 (92.9)	63 (30.3)	93 (44.7)	100 (48.1)	199 (95.7)
≥75	43 (16.6)	62 (23.9)	100 (38.6)	248 (95.8)	55 (51.9)	72 (67.9)	50 (47.2)	98 (92.5)
Median age, yrs (range)	71 (27–95)	65.5 (19–101)	51 (14–101)	48.5 (12–101)	56 (15–100)	50 (12–100)	42 (12–100)	36 (12–100)

**Abbreviations:** ED = emergency department; NA = not applicable; NH = non-Hispanic.

* Health care encounters were defined as hospitalizations, ED visits, outpatient visits, or virtual care visits identified in the period 3 days before through 14 days after the first positive SARS-CoV-2 molecular test date; numbers shown represent the number and percentage of persons with each type of encounter; persons might have received care in multiple encounter settings.

^†^ In-person ambulatory clinic or urgent care visit.

^§^ Telephone or video visit, email messages, online intake, and text chats.

^¶^ Persons who self-identified as Hispanic or Latino ethnicity were categorized as Hispanic. Persons who identified as American Indian, Alaskan Native, Black, African American, Native Hawaiian, Other Pacific Islander, multiracial, or any other race were categorized as non-White, NH. The non-White, NH category most commonly included persons who identified as Asian or Black.

Among 492 hospitalizations, 100 of 117 (85%) that occurred in vaccinated persons and 348 of 375 (93%) in unvaccinated persons were determined to be COVID-19–related after medical record review (Table 3). COVID-19 hospitalizations were rare among fully vaccinated adolescents and young adults; 72% of hospitalizations among fully vaccinated persons occurred in persons aged ≥65 years, (median age = 72 years), 89% of vaccinated persons who were hospitalized because of COVID-19 had at least one underlying medical condition, 15% required ICU admission, and 21 (21%) patients died. In contrast, hospitalizations among unvaccinated persons were more evenly distributed across age groups: 33% were among persons aged ≥65 years (median age = 57 years), 63% had at least one underlying medical condition, 27% required ICU admission, and 58 (17%) died. The median age at death was 78 years (range = 54–94 years) among fully vaccinated and 68 years (range = 37–100 years) among unvaccinated hospitalized patients.

**TABLE 3:** Characteristics of COVID-19–associated hospitalizations* among vaccinated and unvaccinated persons — Oregon and Washington, July 4–September 25, 2021

Characteristic	No. (%)	
	Vaccinated*	Unvaccinated*
**Overall**	**100 (100)**	**348 (100)**
**COVID-19 vaccine product**		
Janssen (Johnson & Johnson)	18 (18.0)	NA
Pfizer-BioNTech	71 (71.0)	NA
Moderna	11 (11.0)	NA
**Race/Ethnicity^†^**		
White, NH	79 (79.0)	249 (71.6)
Non-White, NH	10 (10.0)	49 (14.1)
Hispanic	9 (9.0)	30 (8.6)
Unknown	2 (2.0)	20 (5.7)
**Sex**		
Female	54 (54.0)	144 (41.4)
Male	46 (46.0)	204 (58.6)
**Age group, yrs**		
12–17	0 (—)	≤5 (—)
18–24	0 (—)	≤5 (—)
25–34	≤5 (—)	27 (7.8)
35–44	≤5 (—)	37 (10.6)
45–54	8 (8.0)	85 (24.4)
55–64	16 (16.0)	75 (21.6)
65–74	33 (33.0)	62 (17.8)
≥75	39 (39.0)	55 (15.8)
Median age, yrs (range)	72 (27–95)	57 (16–100)
**Underlying medical conditions**		
≥1 underlying condition (among those listed here)	89 (89.0)	219 (62.9)
BMI ≥30	59 (59.0)	157 (45.1)
Diabetes mellitus (Type I or II)	24 (24.0)	98 (28.2)
Chronic kidney disease	32 (32.0)	37 (10.6)
COPD	24 (24.0)	22 (6.3)
Dementia	10 (10.0)	15 (4.3)
Solid organ transplant	≤5 (—)	≤5 (—)
**Hospital course and outcome^§^**		
Mean length of stay, days (SD)	7.4 (5.7)	9.5 (9.6)
Median length of stay, days (range)	6 (1–31)	6 (1–66)
Intensive care unit admission	15 (15.0)	94 (27.0)
Intubation	8 (8.0)	56 (16.1)
Mechanical ventilation	≤5 (—)	30 (8.6)
Death^¶^	21 (20.2)	58 (17.1)
Median age at death, yrs (range)	78 (54–94)	68 (37–100)

**Abbreviations:** BMI = body mass index; COPD = chronic obstructive pulmonary disease; NA = not applicable; NH = non-Hispanic.

* Excludes 17 of 117 hospitalizations among vaccinated persons and 27 of 375 among unvaccinated persons that were determined after medical record review to be unrelated to COVID-19.

^†^ Persons who self-identified as Hispanic or Latino ethnicity were categorized as Hispanic. Persons who identified as American Indian, Alaskan Native, Black, African American, Native Hawaiian, Other Pacific Islander, multiracial, or any other race were categorized as non-White, NH. The non-White, NH category most commonly included persons who identified as Asian or Black.

^§^ Includes nine ongoing hospitalizations at the time of reporting; length of stay and death data for these hospitalizations are incomplete; these patients are included in the intensive care unit, intubation, and mechanical ventilation totals.

^¶^ Death counts exclude persons with ongoing hospitalization at the time of the final data pull (one vaccinated and eight unvaccinated persons). Death counts also exclude two deaths (one vaccinated and one unvaccinated person) that occurred without hospitalization. These two deaths are included in the crude mortality rate reported in the text.

## Discussion

Previous studies have demonstrated that symptomatic COVID-19 requiring emergency care and hospitalization was uncommon in fully vaccinated persons before widespread circulation of the SARS-CoV-2 Delta variant (*6*,*7*). Incidence among fully vaccinated persons during the period of Delta predominance was approximately three times lower than that in unvaccinated persons across all sex, race, ethnicity, and age groups evaluated. In addition, fully vaccinated persons with SARS-CoV-2 infection were one half as likely to have an ED visit or hospitalization as were unvaccinated patients. Among those hospitalized, vaccinated patients were older than unvaccinated patients, and a higher percentage had at least one underlying medical condition. The crude risk for COVID-19–related death in fully vaccinated persons was sevenfold lower than that among unvaccinated COVID-19 patients. These findings are consistent with another recently published report regarding COVID-19 incidence during Delta circulation, which showed that vaccination is protective against severe illness from COVID-19 (*8*).

The findings in this report are subject to at least six limitations. First, some persons might have received COVID-19 vaccines outside of KPNW (e.g., at a mass vaccination site) and might have been misclassified as unvaccinated if the record was not available in the EMR or immunization information system. Second, persons who had a positive SARS-CoV-2 rapid antigen or other test result at home, school, or the workplace might have been missed, and information about previous SARS-CoV-2 infection was not collected. Third, race and ethnicity were unknown in >10% of the study population, and multiple racial groups were combined into a non-White, non-Hispanic category for some analyses to address small sample sizes. Fourth, medical encounters other than hospitalizations among persons with SARS-CoV-2 infections were not manually reviewed to determine whether symptoms, diagnoses, and treatments were consistent with COVID-19. It is not possible, therefore, to classify all identified infections as symptomatic or asymptomatic. Fifth, the crude rates reported in this report were not adjusted for factors that could influence the risk for infection between the vaccinated and unvaccinated groups. Finally, information about length of hospital stay and death was unavailable for nine hospitalizations that were ongoing at the time of this report.

During this period of widespread Delta variant circulation (July–September, 2021), incidence of SARS-CoV2 infections was lower in fully vaccinated persons and was less likely to result in an ED visit, hospitalization, or death compared with cases in unvaccinated persons. These data support CDC recommendations for COVID-19 vaccination, including additional and booster doses, for the public to protect itself against severe COVID-19, including illness and hospitalization caused by the Delta variant. CDC currently recommends that all persons aged ≥5 years should be fully vaccinated against COVID-19 to prevent illness and reduce transmission of SARS-CoV-2 (*1*).

**Summary**

**What is already known about this topic?**

Studies have demonstrated that SARS-CoV-2 infection, need for emergency department (ED) visits, and hospitalization were uncommon in fully vaccinated persons before the widespread circulation of the SARS-CoV-2 B.1.617.2 (Delta) variant.

**What is added by this report?**

Among persons aged ≥12 years enrolled in a Pacific Northwest health plan, unvaccinated persons with SARS-CoV-2 infection were approximately twice as likely to receive ED care or to be hospitalized than were vaccinated persons with COVID-19.

**What are the implications for public health practice?**

The findings in this report support CDC’s current recommendation that all persons aged ≥5 years should receive full COVID-19 vaccination, including additional and booster doses, to prevent illness and reduce transmission of SARS-CoV-2.

## Footnotes

* Two mRNA vaccines authorized for use in the United States include Pfizer-BioNTech (BNT162b2) and Moderna (mRNA-1273). The Janssen (Johnson & Johnson [Ad26.COV2]) COVID-19 vaccine contains double-stranded DNA encoding a variant of the SARS-CoV-2 spike glycoprotein inserted into a replication-incompetent human adenovirus type 26 viral vector.

† 45 C.F.R. part 46, 21 C.F.R. part 56; 42 U.S.C. Sect. 241(d); 5 U.S.C. Sect. 552a; 44 U.S.C. Sect. 3501 et seq.
